# The Effect of Social Media Consumption on Emotion and Executive Functioning in College Students: an fNIRS Study in Natural Environment

**DOI:** 10.21203/rs.3.rs-5604862/v1

**Published:** 2024-12-23

**Authors:** Anna Aitken, Ali Rahimpour Jounghani, Laura Moreno Carbonell, Anupam Kumar, Seth Crawford, Audrey K. Bowden, S.M. Hadi Hosseini

**Affiliations:** 1 Computational Brain Research and Intervention (C-BRAIN) Laboratory, Department of Psychiatry and Behavioral Sciences, School of Medicine, Stanford University, Stanford, California, USA; 2 Department of Bioengineering, Stanford University, Stanford, California, USA; 3 Department of Biomedical Engineering, Vanderbilt University, Nashville, TN, USA; 4 These authors have equally contributed to this work.

**Keywords:** Social Media, fNIRS, Executive Functioning, Emotion, College Students

## Abstract

As of 2023, 69% of adults and 81% of teens in the U.S. use social media. This study explores the immediate effects of social media consumption on executive functioning (EF) and emotion in college students, using a wearable fNIRS system to monitor brain activity in a naturalistic setting. Twenty participants were assessed pre- and post-social media use through EF tasks and emotion questionnaires. Results revealed 55% of participants were classified as addicted, with an average Instagram usage of 5 hours per week. Following social media exposure, significant impairments were observed in tasks like n-back and Go/No-Go, alongside altered brain activity. Specifically, increased medial prefrontal cortex (mPFC) activity indicated heightened cognitive effort and performance monitoring, while decreased dorsolateral prefrontal cortex (dlPFC) and ventrolateral prefrontal cortex (vlPFC) activity were associated with impaired working memory and response inhibition. Inferior frontal gyrus (IFG) activity reductions correlated with difficulties in inhibiting motor responses to No-Go stimuli. Emotional changes were minimal, except for reduced happiness in the control group. These findings highlight the negative impact of social media on EF, emphasizing the need for interventions promoting healthier digital habits.

## Introduction

As of 2023, social media usage is widespread, with 69% of adults and 81% of teens in the U.S. actively engaging with these platforms (Sperling, 2023). A 2023 survey revealed that U.S. teenagers spend an average of 4.8 hours per day on social media platforms, while only 10.5% of teenagers report spending one hour or less on these platforms (Rothwell, 2023). Many individuals exhibit addictive behaviors towards social media akin to a smoker’s unconscious habit of reaching for a cigarette (Macït et al, 2018). Adolescents and young adults, in particular, often display a muscle memory reflex that prompts them to pick up their phones and open a social media app.

While the academic discourse frequently delves into how prolonged social media use impacts adolescent mental health and academic performance, this study shifts the focus to examining the immediate effects of social media use on brain activity and behavior. The surge in global social media usage among college and university students has sparked discussions about its potential influence on academic performance. However, existing evidence on this connection remains inconclusive. A review conducted by Valkenburg et al. (2022) revealed varying perspectives, with some researchers characterizing the association between mental health issues and social media usage as ‘weak’ or ‘inconsistent,’ while others deemed it ‘substantial’ and ‘deleterious’. Suárez-Perdomo’s study in 2022 identified significant differences in procrastination behaviors linked to social network addiction among students, yet no notable variations were found in academic performance (Suárez-Perdomo et al., 2022). These disparities underscore the complexities in the literature surrounding the impact of prolonged social media use on different facets of well-being.

Executive function (EF) encompasses higher-level cognitive skills that coordinate and control cognitive abilities and behaviors, including inhibition, working memory, and cognitive flexibility. First, inhibitory control involves overriding automatic responses, allowing individuals to change their reactions consciously (Diamond, 2013; Murphy et al., 1999). Second, working memory, essential for reasoning and understanding language, requires individuals to actively hold and manipulate information (Diamond, 2013). Third, cognitive flexibility, emerging later in development, involves shifting perspectives and adapting to new situations (Blair, 2016; Diamond, 2013). EFs are critical for academic success, influencing areas such as problem-solving, learning, planning, and impulse inhibition (Blair, 2016; Diamond, 2013; Doebel, 2020; Logue & Gould, 2014; Ramos-Galarza et al., 2023), but they also play a pivotal role in daily life tasks such as time management, decision-making, and regulating emotions. Impairments in EF can reduce attention span, decrease cognitive abilities, and lower overall productivity (Appel et al., 2020).

Previous neuroimaging studies using tools like EEG and fMRI have provided valuable insights into executive functioning (Collette et al., 2006). However, their application is often constrained by the need for controlled environments and static setups, limiting their ability to assess EF during everyday activities. Our study aims to address this gap by leveraging a wearable functional near-infrared spectroscopy (fNIRS) device. This portable and user-friendly platform allows for the real-time, standardized assessment of EF during naturalistic settings. Unlike traditional approaches, our wearable fNIRS system enables the exploration of neurocognitive processes as they unfold in real-world scenarios, offering an ecologically valid perspective on how individuals manage cognitive demands in their daily routines.

The relationship between specific aspects of EF and distinct regions of the prefrontal cortex (PFC) is well-established. These PFC areas are intricately connected to various brain regions, including other cortical areas and structures associated with emotional reactivity, attention control, and stress response (Blair, 2016; Jahani et al., 2015). While EFs are primarily mediated by prefrontal cortical function, they are also subject to modulation by neurotransmission inputs of dopamine, noradrenaline, serotonin, and acetylcholine. The adaptability of cognitive behavior in response to environmental changes is facilitated by these neurotransmitter systems’ modulating effects on executive function. However, alterations in these systems can significantly impact EF (Blair, 2016; Logue & Gould, 2014).

Dopamine, a neurotransmitter with roles extending beyond EF, is implicated in processes like reward encoding and drug addiction. Reduced dopamine activity in the medial prefrontal cortex (mPFC) is associated with poor performance in set-shifting and attention tasks, while elevated dopamine activity improves these cognitive functions (Logue & Gould, 2014). Norepinephrine activity influences all four cognitive processes supporting executive function. Active in both the mPFC and orbitofrontal cortex (OFC), it regulates overall arousal levels and establishes basal levels of cortical activity. Consequently, arousal levels dictate the activation of various norepinephrine receptor types, contributing to the modulation of cognitive processes. The serotonin system plays a crucial role in the OFC mediating response inhibition. Lower serotonin levels are associated with impaired response inhibition, highlighting the multifaceted influence of neurotransmitter systems on executive function. Finally, the inferior frontal cortex also plays an important role in response inhibition. The right inferior frontal gyrus (IFG) is considered a key node for the inhibition of motor responses, which is a core aspect of impulse control (Boen et al., 2022; Logue & Gould, 2014).

Emerging evidence suggests that social media use may transiently alter neurotransmitter activity, particularly dopamine, due to its rewarding and reinforcing nature. Given the critical role of dopamine, norepinephrine, and serotonin in supporting executive function, such alterations may partially explain the immediate effects of social media on EF. These mechanisms highlight a potential link between the neurochemical changes induced by social media and the observed impairments in cognitive processes like attention and response inhibition.

A recent study used fNIRS to examine the impact of tablet use on preschoolers’ executive function in lab environment. The device revealed distinctive patterns in the PFC between heavy tablet users and non-users (Li et al., 2021). Among the 30 tablet users, 16 were classified as *heavy users* based on their daily screen time exceeding the mean level (M = 17.98 min, SD = 14.29), uncontrolled or unlimited tablet use, and engagement in multiple activities on the tablet. The remaining 14 users, classified as *low users*, were excluded from the comparative analysis. The study incorporated an EF measure, specifically the Dimensional Change Card Sort (DCCS) task, which assesses cognitive flexibility, concurrently with the fNIRS tool. The results revealed that *heavy users* exhibited lower performance on the DCCS task compared to *non-tablet users*, indicating better EF performance among the latter group. Moreover, the activation patterns in the prefrontal cortex varied significantly between the two groups. Interestingly, the pattern observed in the ‘non-user’ group aligned with expectations and was considered typical. In contrast, the pattern observed in the ‘heavy-user’ group was unexpected. The heavy-user pattern was characterized by a significant decrease in oxygenated hemoglobin concentration (Oxy-Hb) and an increase in deoxygenated hemoglobin concentration (deOxy-Hb) in the PFC. Finally, this exemplifies the feasibility of using fNIRS to explore the neural underpinnings of EF in the context of modern technology use, such as social media engagement in lab environment.

We aimed to pioneer the use of our wearable fNIRS system (Tsow at al., 2021) to examine the immediate effects of social media consumption and its implications for EF and emotion. The low-cost, portability, and ecological validity of our wearable fNIRS platform are highly valuable and enable research to be done regardless of location or expense. This approach represents a novel contribution to the existing body of research as it explores real-time neurocognitive and emotional responses to social media use, particularly among the demographic most immersed in these platforms.

Our study aims to investigate how social media consumption affects PFC activity during cognitive tasks, using our wearable fNIRS system in a natural setting. By “natural setting,” we refer to an environment that closely resembles everyday conditions rather than a traditional, controlled laboratory. In this case, all data were collected in a quiet, private room in a student residence building, which allowed participants to engage in social media use in a context that mirrors typical, real-world interactions with technology. This approach enhances ecological validity by capturing brain activity in settings that participants are more accustomed to, providing a more accurate reflection of how social media impacts cognitive functioning during daily life.

Our approach provides the flexibility to monitor brain activity while participants engage in everyday behaviors. In this study, we explored the impact of social media use on EF and emotional state by examining behavioral performance and correlating it with prefrontal cortical activity. Participants were divided into two groups: a control group and a social media use group. Both groups completed EF tasks, such as working memory and inhibition exercises, at two sessions—before and after engaging in their respective activities.

Using fNIRS, we measured prefrontal activity via oxygenated hemoglobin levels. Based on our hypothesis, we anticipated a group-by-session interaction, where participants in the social media use group would show increased prefrontal activity in regions associated with EF, such as working memory and inhibition, relative to the control group. These results would provide insight into the neural correlates of social media’s impact on cognitive and emotional processing. Furthermore, we predict that social media use would result in a decline in positive emotions and impairments in executive function. The findings of this study offer valuable insights into the influence of social media on cognitive processes and its potential implications for cognitive health, particularly in relation to executive functioning.

## Results

Table 1 depicts the social media usage trends among the participant population. Participants were asked to report their weekly Instagram usage and complete a questionnaire regarding their relationship to social media. The Social Media Addiction Scale (SMAS) results revealed that % of participants are classified as addicted to social media (threshold for classification of addiction was a score of >90). It also revealed that participants on average used Instagram for 5 hours/week, although this did not account for screen time on other social media platforms.

Our study included 20 participants, divided into a control group (*n*_*social Media group*_:10) and a social media use group (*n*_*Control group*_ :10). Both groups completed EF tasks, such as working memory and inhibition exercises, at two sessions—before and after the intervention.

### Behavioral Findings

We investigate whether the EF task measurements showed differences in behavioral performances on the EF tasks between groups before and after the intervention (more info in the Methods section). The experimental, social media group viewed their Instagram following page, whereas the control group were exposed to black and white nature photos, which are more neutral and emotionally uncharged.

**Figure 1.**
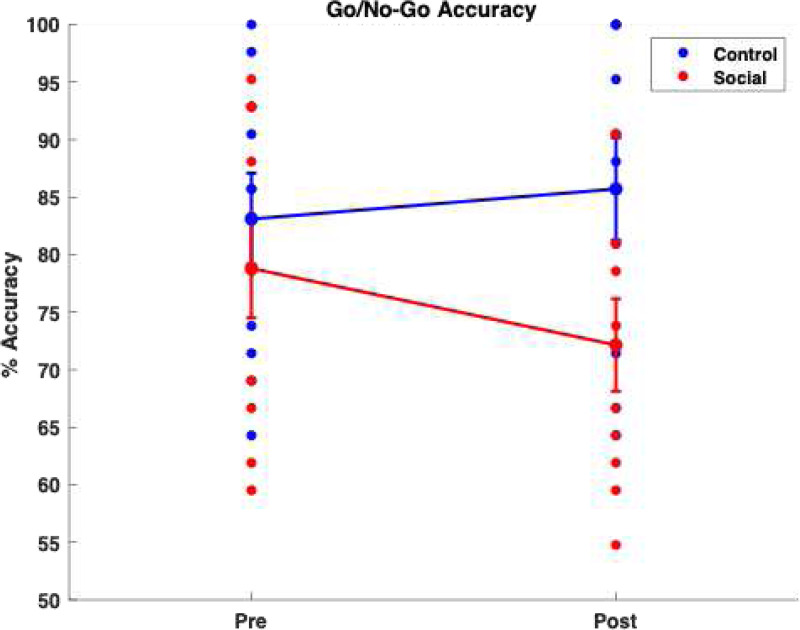
Significant group by session interaction effect (p-value = 0.0017) in Go/No-Go behavioral accuracy

As shown in Figure 1, the social media group demonstrated significantly poorer performance compared to the control group (*p*_*adjusted*_ = 0.012, *r* = −0.66), despite exhibiting similar reaction times (Figure S1). This finding supports the hypothesis that executive function task performance declines following social media use. Additionally, on the memory task (3-back), the social media group exhibited a nonsignificant trend toward poorer performance relative to the control group after social media use (*p*_*adjusted*_ = 0.17, Figure S2). However, a high negative effect size (Rank-Biserial Correlation, *r* = −0.55) was observed, indicating a meaningful practical difference in change memory scores between groups.

The results of the Discrete Emotions Questionnaire (DEQ) revealed that other than in the only observed in the Happiness Items Score, there was no significant change in emotions following social media use. The analysis revealed no statistically significant group-by-session interaction effect on the Happiness Items of the DEQ (*p*_*adjusted*_ = 0.1163); moreover, a medium negative effect size (Rank-Biserial Correlation, *r* = −0.2667) indicated a practical difference in change scores between groups (Figure S3).

### fNIRS Findings

**Figure 2.**
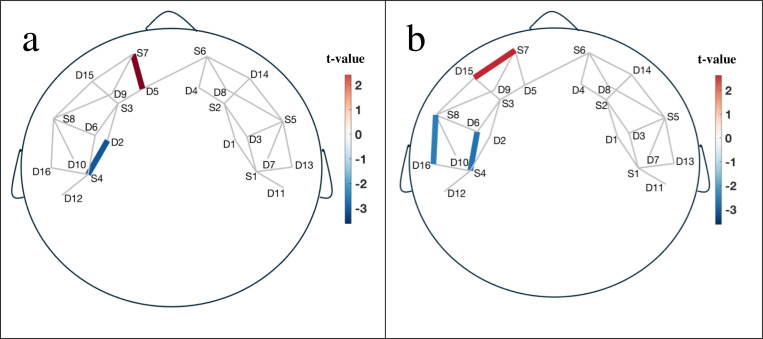
Channel maps of group and session interaction effect showing significant cortical Oxy-Hb activation in the left prefrontal areas during a) n-back task and b) Go/No-Go task. The color bar represents t-value at those locations.

Significant interaction effect between group and session were found in the fNIRS activation patterns during two executive functioning tasks (n-back and Go/No-Go tasks). Figure 2 shows the left-lateralized mPFC, dorsolateral prefrontal cortex (dlPFC), ventrolateral prefrontal cortex (vlPFC), IFG activation. During the n-back task (Figure 2a), increased activity in the mPFC (t_S7−D5_ = 3.32, p < 0.05, Cohen’s d = 1.1; Refer to Figure 5b for the corresponding source-detector pairs layout) is often related to the monitoring of ongoing performance and high regulation of cognitive effort in social media usage comparing to control group. During the Go/No-Go task (Figure 2b), the mPFC (t_S7−D15_ = 3.13, p < 0.05, Cohen’s d = 1.4) is involved in monitoring actions and detecting errors. Increased mPFC activity is associated with high effort to inhibit responses and performance monitoring influenced by social media.

The dlPFC is crucial for maintaining and manipulating information in working memory (Barbey et al., 2013). Decreased activity in the dlPFC (t_S4−D2_ = −3.4, p < 0.05, Cohen’s d = 0.8) during the n-back task reflects less cognitive load to update and maintain the working memory representations. The dlPFC is also engaged in maintaining task rules and exerting top-down control to inhibit inappropriate responses. Decreased activity in the dlPFC (t_S4−D6_ = −2.7, p < 0.05, Cohen’s d = 0.9) during the Go/No-Go task is associated with the unsuccessful inhibition of responses to No-Go stimuli affected by social media.

The vlPFC is involved in response inhibition and conflict resolution (Egner, 2011). Decreased activity in the vlPFC (t_S8−D16_ = −2.5, p < 0.05, Cohen’s d = 1.3) during the Go/No-Go task reflects low cognitive effort to inhibit responses and a failure to resolve conflicts between Go and No-Go signals. Finally, the IFG is critically involved in inhibitory control. Decreased activity in the IFG during the Go/No-Go task (t_S8−D16_ = −2.5, p < 0.05, Cohen’s d = 1.3) is strongly associated with the failure to suppress motor responses and withhold actions in response to No-Go stimuli resulted from social media.

## Discussion

In 2023, with 6.92 billion smartphone users worldwide, social media applications such as Instagram, Twitter, TikTok, and Facebook dominate the digital landscape, influencing approximately 86% of the global population (Zhang et al., 2023). Our study contributes to the growing body of research exploring the complex relationship between social media use and EF. Utilizing a wearable, portable fNIRS system in a naturalistic setting, this study provides novel insights into how social media consumption affects cognitive functioning and emotional states in college students, not only within academic settings but also in their everyday life situations, such as social interactions, time management, and decision-making.

In our study, high level of social media engagement was associated with performance differences on Go/No-Go task. Specifically, participants in the social media group exhibited lower accuracy compared to the control group. These findings suggest that prolonged social media consumption may affect cognitive functions like working memory and inhibitory control, although further analysis is required to confirm the extent of these associations

Our fNIRS data provided a more detailed view of these impairments by highlighting specific changes in brain activity. We observed increased activity in the mPFC during n-back and Go/No-Go tasks, which is typically associated with heightened cognitive effort and performance monitoring while social media usage. This suggests that participants may have exerted more effort to maintain their performance on these tasks after using social media, possibly as a compensatory response to the cognitive demands imposed by social media engagement. Conversely, decreased activity resulted from social media use was observed in the dlPFC and vlPFC, which are regions critical for maintaining working memory and exerting response inhibition, respectively. The reduction in activity in these areas indicates a diminished capacity to effectively manage working memory and inhibit inappropriate responses, reinforcing the idea that social media use can disrupt normal executive functioning.

The findings of our study align with a significant body of literature that highlights the negative impact of media multitasking on cognitive abilities, including attention and memory (Baumgartner et al., 2014; Kobayashi et al., 2020; Madore et al., 2020). However, our study advances the field by being the first to measure these effects using fNIRS in a natural setting, providing a level of ecological validity that many laboratory-based studies lack.

Our results also support the notion of a bidirectional relationship between social media use and EF. Previous studies have suggested that strong EF can act as a buffer against addictive behaviors, while a decrease in inhibitory control can exacerbate behaviors such as excessive social media use (Wegmann et al., 2020). However, the literature also presents some inconsistencies, particularly regarding the impact of social media use on attention in academic settings and its effects on academic performance. Some studies report no significant changes or even improvements in EF following exposure to certain types of social media content, particularly those that evoke strong emotional responses (Mayshak et al., 2016). However, our findings align with prior research by Lench et al. (2011), which suggests that happiness specifically impacts cognition. It may also be that participants found the control condition less engaging and therefore were less likely to report being happy. These inconsistencies suggest that the nature of the content, individual differences in emotional regulation, and the context of social media use are critical factors that may influence the outcomes.

The implications of our findings are multifaceted, particularly in the realms of cognitive health, education, and social behavior. Firstly, the observed decline in EF suggests potential repercussions for individuals’ cognitive health. Impairments in planning, inhibition, and cognitive flexibility could impede daily tasks, compromise focus, and lead to suboptimal decision-making, which, in turn, could impact overall mental well-being (Wang & Tchernev, 2012).

Secondly, these cognitive impairments have direct implications for academic and professional success. Students and professionals rely heavily on their executive functions for effective learning, problem-solving, and task management. A decline in these cognitive abilities, as observed following social media use, could negatively affect academic achievement, reduce productivity, and potentially hinder long-term career prospects, thereby impacting economic outcomes and societal progress (Blair & Razza, 2007; Duckworth & Seligman, 2005).

Moreover, the social ramifications of diminished EF should not be overlooked. Effective social interactions require proficient EFs, including empathy, impulse control, and perspective-taking. A decline in these areas could lead to challenges in understanding others’ perspectives, regulating emotions, and engaging in meaningful communication. This could adversely affect the quality of relationships, exacerbate social conflicts, and hinder the development of crucial social skills, particularly among younger individuals who are heavily immersed in social media (Carlson & Moses, 2001; Denham et al., 2003; Eisenberg & Spinrad, 2004).

Given the significant implications of our findings, there is a clear need for targeted interventions at both the policy and educational levels. Policymakers should consider implementing regulations that encourage healthy online behaviors and promote a balanced approach to digital engagement. Initiatives aimed at improving digital literacy, protecting privacy, and ensuring online safety should also include strategies to support cognitive well-being and mitigate the risks associated with excessive social media use. Similarly, educational strategies should integrate digital literacy programs that emphasize self-regulation, critical thinking, and the development of cognitive skills such as impulse control and inhibitory function. Educators and parents should be made aware of the potential cognitive consequences of social media use and encouraged to foster environments that promote mindful and balanced digital consumption.

While our study provides valuable insights, it is important to acknowledge its limitations. The small sample size and focus on college students may limit the generalizability of our findings to broader populations. Additionally, the fNIRS device, while effective for measuring cortical activity, does not capture activity in deeper brain structures that may influence executive functioning. Future research should aim to replicate these findings in larger and more diverse populations to enhance generalizability. Investigating the long-term effects of chronic social media exposure and isolating specific aspects of consumption—such as content type and usage context—could further clarify its distinct impacts on cognitive performance and emotional regulation.

Moving forward, research should also explore interventions to promote healthier digital consumption habits. Tools such as digital detox programs or self-regulation techniques could help mitigate the negative cognitive effects of social media use. Moreover, studying the impact of different types of social media content on EF and emotional regulation would provide valuable insights into the nuanced ways digital habits shape cognitive health over time.

## Conclusion

This study highlights the immediate effects of social media consumption on executive functioning in college students, demonstrating its impact on cognitive processes like working memory and inhibition, as measured by fNIRS. Increased activity in the mPFC after social media use suggests heightened cognitive effort and performance monitoring, while decreased activation in the dorsolateral and ventrolateral PFC reflects impairments in working memory and response inhibition. Additionally, reduced activity in the IFG, a region critical for inhibitory control, was associated with difficulties in suppressing motor responses to No-Go stimuli. These findings underscore the importance of understanding how digital consumption affects cognitive health.

## Methods

### Participants

Participants were gathered via a recruitment flyer posted across campus. A pool of 20 undergraduate students (11 women and 9 men; mean age ± SD= 20.75±1.164) was randomly recruited (Table 2). Participants were then randomly assigned to either a control group (i.e., who scrolling through neutral photos on social media) or experimental group (i.e., who scrolling through social media). None of the participants reported any neurological disorder or injury that would prevent them from performing executive functioning tasks. This study was approved by the Stanford University School of Medicine Institutional Review Board for research ethics and human participants. Informed consent was obtained from all twenty individual participants.

All methods were performed in accordance with relevant guidelines and regulations, as approved by the Stanford University School of Medicine Institutional Review Board for research ethics and human participants. In addition, informed consent was obtained from all participants for participation in the study and publication of any identifying information or images in an online open-access publication. All identifying details have been excluded from the submitted materials.

### Experimental Procedures

**Figure 3.**
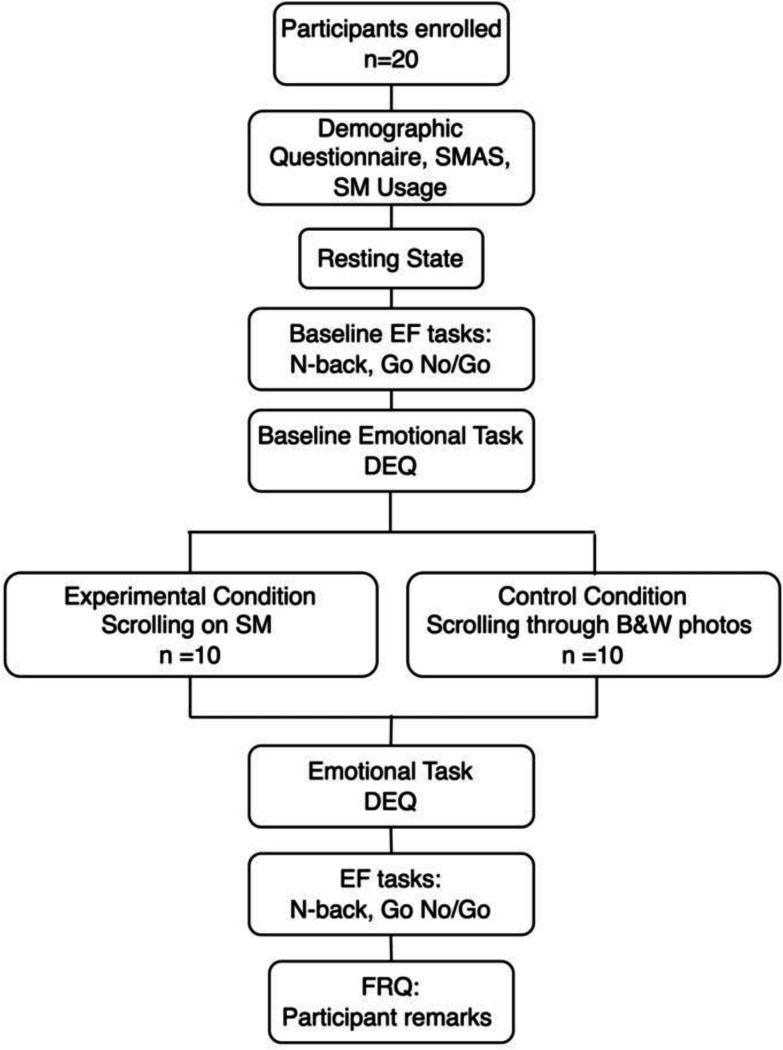
Procedure flow chart

The detailed procedure is presented in Figure 3. Data collection was completed in one 80-minute session after participants completed a consent form and were re-screened for exclusion criteria. Upon arrival, participants filled out basic demographic information and completed the SMAS and a questionnaire about general social media consumption (e.g., screen time, app type). They then put on the wearable fNIRS headband and recorded the basal brain activity at rest, establishing a baseline reading, before conducting the first round of EF tasks and emotion tasks.

The resting state condition established the baseline brain activity specific to the person. Participants were instructed to close their eyes and relax for 7 minutes. Following this, emotional and EF tasks were performed to establish a baseline performance before the intervention (social media use) or control condition. After this, the participant scrolled on their Instagram “following” page for 15 minutes. A 15-minute session allowed time for collecting enough fNIRS data, while still mimicking a typical social media session. Following this, the same assessments were performed post-social media use to compare results with the baseline. The control group underwent the same procedure but scrolled through a curated selection of black-and-white nature images with captions to mimic the same kinds of visual inputs without the same emotionality associated with them.

### Outcome Measures and Experimental Procedure

This study employs a mix of objective and subjective measures. The objective measures include standardized, validated computer-based tasks targeting various facets of EF, such as working memory and inhibition. Subjective measures involve self-report and informant-report questionnaires designed to evaluate emotion regulation in participants’ daily life situations.

### Objective Measures

#### N-back Task:

**Figure 4.**
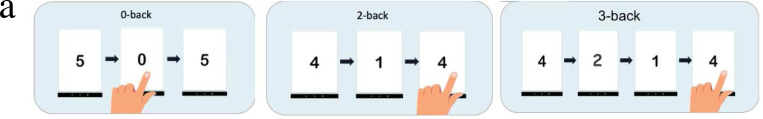

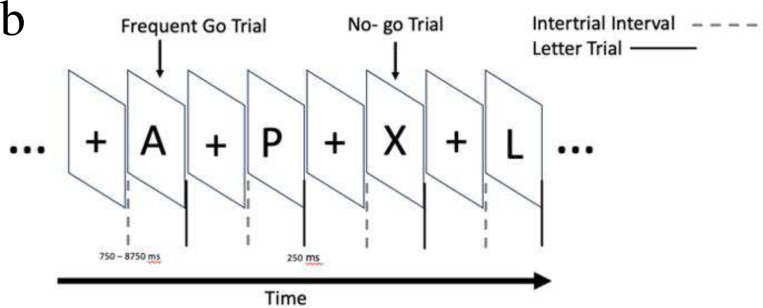
Designed cognitive tasks represented in tablet app given names as a) n-back and b) Go/No-Go tasks.

This task measures working memory. Participants monitor a series of stimuli (i.e., numbers) and indicate whether the current stimulus matches the one presented n steps back in the sequence (Figure 4a). The task includes three levels (0-back, 2-back, 3-back), each repeated four times. Participants tap the screen based on specific number sequences. The difficulty of the task is modulated by altering the value of n. Outcome measures include accuracy and reaction time, offering insights into participants’ working memory performance.

#### Go/No-Go Task:

Designed to assess inhibition, this task (Shown in Figure 4b) presents a series of stimuli (i.e., letters) prompting participants to respond to one type of stimulus (Go) while inhibiting their response to another type of stimulus (No-go). A total of 420 trials were presented, consisting of go (30%), no-go (10%), and non-trials (60%). Participants tapped for specific alphabet letters (go trials) and avoided tapping for the letter X (no-go trial), with non-trials (displayed as blank) introducing jittering. Outcome measures encompass accuracy and reaction time, providing valuable information on participants’ ability to suppress automatic responses and exert control over attention.

### Subjective Measures

#### Discrete Emotions Questionnaire (DEQ):

The DEQ is sensitive to eight distinct state emotions: anger, disgust, fear, anxiety, sadness, happiness, relaxation, and desire. Participants answer questions like: “While [doing this action], to what extent did you feel [emotion]?” They rank their experience from 1 (not at all) to 7 (an extreme amount). The outcome measure is the sum of the scores indicated in each of the eight categories.

#### Social Media Addiction Scale (SMAS):

The SMAS measures dependence on social media. It reflects six features of addiction proposed in Griffiths’s components model of addiction: salience, mood modification, tolerance, withdrawal, conflict, and relapse. Participants rank how they relate to statements from 1 (very rarely) to 5 (very often). The outcome measure is the sum of the scores of all questions.

### Neuroimaging

**Figure 5.**
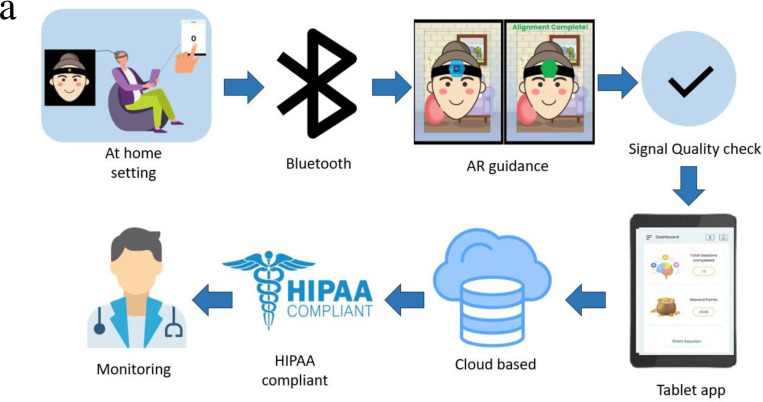

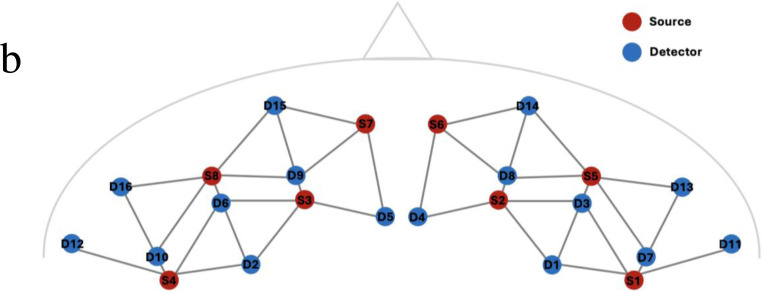
a) Wearable fNIRS platform for measuring brain activity in natural setting and b) corresponding source-detector pairs layout

The study used a custom-built fNIRS platform, a wearable, wireless, multi-channel, smartphone-operated optical imaging system for monitoring prefrontal activity (Figure 5). It consists of 7 light sources and 16 detectors arranged on 33 regular and 4 short channels, covering the forehead area and measuring prefrontal cortex activity involved in executive function. The device operates at two wavelengths of light (740 nm and 850 nm) and has a sampling rate of 7 Hz. It connects to a tablet running software for data acquisition and analysis, and it is wireless and battery-powered, allowing for mobility and flexibility in the experimental setting (Tsow et al., 2021).

### Data Analysis

The preliminary phase of analyzing fNIRS data included a rigorous quality control procedure to pinpoint and eliminate channels of inferior quality. Channels with a Signal Noise Ratio (SNR) and Quality Index (QI) below set thresholds (SNR < 20 dB and QI < 0.5) were discarded. Baseline corrections were made to the raw intensity signals to address issues like direct current shifts, concatenation effects, and global signals (Rahimpour Jounghani et al., 2023). Motion artifacts were tackled using wavelet filtering (Hosseini et al., 2017) with a sym8 wavelet, discarding data points that deviated by more than five standard deviations from the mean, primarily focusing on motion and very low-frequency disturbances. The raw data were then used to extract levels of Oxy-Hb and deOxy-Hb using the modified Beer-Lambert law.

The AR-IRLS regression model by Santosa et al. (2020), implemented via the BrainAnalyzIR toolbox (Santosa et al., 2018), improved signal fidelity and identified regressors by utilizing data from short channels, reducing physiological and additional motion-related artifacts and enhancing fNIRS measurement reliability (Gagnon et al., 2012).

A general linear mixed regression model was used for further statistical analysis to investigate the influence of different experimental tasks (N-Back and Go/No-Go tasks) on cortical activation. The dependent variables represented by beta (β) corresponded to the effects for Oxy-Hb and deOxy-Hb under each condition. The Benjamini-Hochberg correction method based on the False Discovery Rate (FDR) was applied to reduce the risk of false positives. Moreover, behavioral measures were analyzed using nonparametric statistical tests to account for the small sample size and ordinal nature of the data. Wilcoxon signed-rank tests were used for within-group comparisons (pre- vs. post-intervention), while Mann-Whitney U tests were employed for between-group comparisons of change scores. Multiple comparisons were corrected using the Holm-Bonferroni method to control the family-wise error rate. All statistical analyses were conducted using MATLAB R2022b.

## Supplementary Material

Supplement 1

## Figures and Tables

**Table 1.** Social media addiction measurements

Sample Characteristics	n	%	Average	SD	Max	Min

Time spent on Instagram (hr/week)			4.99	4.4	20	0.5
SMAS Sum Score	20		92.65	20.18	121	60
Participants classified as addicted	11	55				

**Table 2.** Sociodemographic Information

	Social Media (N=10)	Control (N=10)	*Statistics*

Age, mean (SD)	21.75 (1.23)	21.81 (1.11)	*p* = 0.77
Sex, female	5	6	*p* = 0.9
Race, Asian; Black/African American; White	0,3,7	2,3,5	*p* = 0.6
Ethnicity, Latino/Hispanic	5	5	*p* = 1

## Data Availability

The data that support the findings of this study are available from the corresponding author, **ARJ**, upon request (email: rahimpur@stanford.edu)
