# Supplementary material for: The Effect of Social Media Consumption on Emotion and Executive Functioning in College Students: an fNIRS Study in Natural Environment
Source: Res Sq. 2024 Dec 23:rs.3.rs-5604862. Preprint. [Version 1] doi: 10.21203/rs.3.rs-5604862/v1 (PMC11703342; doi:10.21203/rs.3.rs-5604862/v1)
Supplement: Supplement 1 [file NIHPPRS5604862v1-supplement-1.pdf]

Supplementary Files

This is a list of supplementary files associated with this preprint. Click to download.

- SocialMediaSMfinal.docx
